# Evaluation of Crystalline Volume Fraction of Laser-Annealed Polysilicon Thin Films Using Raman Spectroscopy and Spectroscopic Ellipsometry

**DOI:** 10.3390/mi12080999

**Published:** 2021-08-22

**Authors:** Jeongsang Pyo, Bohae Lee, Han-Youl Ryu

**Affiliations:** Department of Physics, Inha University, Incheon 22212, Korea; vywjdtkd789@naver.com (J.P.); bbosea@inha.edu (B.L.)

**Keywords:** laser anneal, crystallization, low-temperature poly silicon, Raman spectroscopy, ellipsometry

## Abstract

We investigated the crystallinities of poly silicon (poly Si) annealed via green laser annealing (GLA) with a 532-nm pulsed laser and blue laser annealing (BLA) with 450-nm continuous-wave lasers. Three-dimensional heat transfer simulations were performed to obtain the temperature distributions in an amorphous silicon (*a*-Si) thin film, and GLA and BLA experiments were conducted based on the thermal simulation results. The crystallinity of annealed poly Si samples was analyzed using Raman spectroscopy and spectroscopic ellipsometry. To evaluate the degree of crystallization for the annealed samples quantitatively, the measured spectra of laser-annealed poly Si were fitted to those of crystalline Si and *a*-Si, and the crystal volume fraction (*f_c_*) of the annealed poly Si sample was determined. Both the Raman spectroscopy and ellipsometry showed consistent results on *f_c_*. The *f_c_* values were found to reach >85% for optimum laser power of GLA and BLA, showing good crystallinity of the laser-annealed poly Si thin films comparable to thermal furnace annealing.

## 1. Introduction

Laser annealing has been widely adopted as a low-temperature polycrystalline silicon (LTPS) process to crystalize amorphous silicon (*a*-Si) materials into polycrystalline silicon (poly Si) grains. Poly Si films with high mobility can be achieved by the LTPS process, leading to the realization of low-power thin-film transistor (TFT) panels in liquid crystal or organic light-emitting diode displays [1,2]. In the laser annealing process, the temperature increase is limited only to a low irradiated volume of a sample, which facilitates an extremely localized LTPS process in microtechnology [3,4,5]. Recently, the use of laser annealing has attracted considerable attention for three-dimensional (3-D) sequential architectures in semiconductors, where the thermal budget for annealing processes is drastically limited [6,7].

In the laser annealing process for a large-area TFT panel, excimer laser annealing (ELA) using ArF (197 nm), KrF (308 nm), or XeCl (308 nm) lasers have been widely employed owing to their high laser power and large absorption coefficient for *a*-Si [8,9,10,11]. However, ELA has the issues of high maintenance cost and material non-uniformity. In recent years, various laser annealing technologies have been developed for LTPS processes as alternatives to ELA: green laser annealing (GLA) [12,13,14,15], near-ultraviolet laser annealing (NULA) [16,17,18,19], and blue laser annealing (BLA) [20,21,22]. GLA and NULA usually employed *Q*-switched diode-pumped solid-state (DPSS) lasers emitted at 532 and 355 nm, respectively. In BLA, high-power continuous-wave (CW) blue laser diodes emitted around 450 nm have been used. DPSS and diode lasers have the advantages of high stability and low maintenance as well as installation costs over excimer lasers. Up until now, good crystallization properties have been reported in poly Si films fabricated using GLA, NULA, and BLA. However, the degree of crystallization has not been quantitatively analyzed in laser-annealed poly Si samples. Therefore, it was not easy to compare the crystal quality of laser-annealed poly Si to that of poly Si samples fabricated by other annealing processes or poly Si samples annealed with different laser sources.

In this paper, we report a quantitative evaluation of the crystallinities of laser-annealed poly Si thin films using Raman spectroscopy and spectroscopic ellipsometry. A 100-nm-thick *a*-Si film deposited on a SiO_2_/Si wafer was used for laser annealing of this study. First, the temperature distributions during laser annealing processes were investigated using a thermal simulation with the COMSOL Multiphysics program to determine the conditions of optimum laser power [23]. Based on the simulation results, laser annealing experiments were conducted on the *a*-Si film using GLA and BLA. In addition, thermal furnace annealing was performed to compare the crystallinities of the laser-annealed samples. Although furnace annealing for *a*-Si is known to show good crystallinity, it requires a high temperature and long process time.

Subsequently, the degree of crystallization for the laser-annealed and furnace-annealed samples was analyzed using Raman spectroscopy and spectroscopic ellipsometry. These are nondestructive optical methods that have frequently been employed to analyze the characteristics of annealed poly Si. Spectroscopic ellipsometry can provide information on the optical constants and film thickness of a poly Si layer [24,25,26]. Raman spectroscopy has the advantages of a simple measurement setup and short measurement time. It has been adopted for the quantitative analysis of crystallinity of poly Si samples fabricated by thermal annealing [27,28,29].

Quite recently, we reported the comparison of the crystallinity of poly Si annealed via NULA, BLA, and GLA at one specific laser power level using the spectroscopic ellipsometry [30]. However, few studies have investigated Raman spectroscopy for the quantitative analysis of the crystallization by laser annealing. In this work, for quantitative evaluation of crystallization, the Raman and optical constant spectra of annealed samples were fitted to those of crystalline silicon (*c*-Si) and *a*-Si, and the crystal volume fraction (*f_c_*) of the annealed poly Si samples was determined for three different laser power levels. It will be revealed that Raman spectroscopy showed consistent results with spectroscopic ellipsometry for all laser power levels, implying that Raman spectroscopy can be conveniently and reliably employed for evaluating the crystal quality of laser-annealed poly Si. 

## 2. Methods

### 2.1. Thermal Simulation

**Figure 1:**
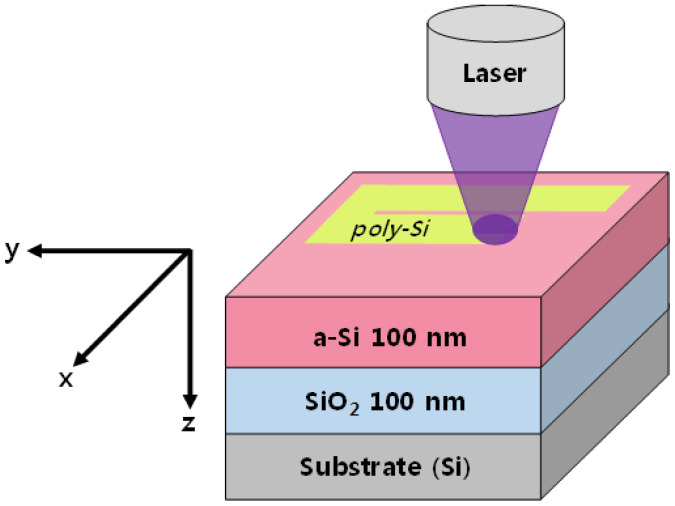
Schematic of the laser annealing experiment for an *a*-Si/SiO_2_ thin film on a single crystalline silicon wafer. The *x*-, *y*-, and *z*-axes represent the Cartesian coordinate system for heat conduction simulations. The laser beam scans the sample surface in the *y* direction.

Figure 1 schematically shows the *a*-Si sample structure and the laser annealing experiment performed in this study. The sample structure consisted of 100-nm-thick *a*-Si and SiO_2_ layers prepared via plasma-enhanced chemical vapor deposition (PECVD) on a silicon substrate. The substrate temperature for PECVD was 200 °C. After the deposition of the *a*-Si film, de-hydrogenation was performed at 500 °C for 30 min to remove hydrogen content in *a*-Si. During the laser annealing experiment, a laser beam was focused on the sample surface using an objective lens and the beam scanned the sample surface in the *xy* plane, as shown in Figure 1. The temperature distribution during the laser illumination was investigated using thermal simulations to determine the optimum laser power level in GLA and BLA processes.

In a non-uniform isotropic medium, three-dimensional (3-D) temperature distribution can be obtained by solving the following heat conduction equation:

(1) $$\rho C_{p}\frac{\partial T}{\partial t}=\nabla \cdot (\kappa \nabla T)+Q(x,y,z,t),$$

where *ρ* is the mass density, *C_p_* is the specific heat capacity, *T* is the temperature, *κ* is the thermal conductivity, and *Q*(*x*, *y*, *z*, *t*) is the volumetric heat source [31,32,33]. Variables *x*, *y* and *z* represent the axes of the coordinate system shown in Figure 1, and *t* is the time. In this study, the heat conduction equation in Equation (1) was solved numerically using the COMSOL Multiphysics program [23].

When the laser light is absorbed by the sample, the light intensity decreases exponentially with the propagation distance. Therefore, according to the Beer-Lambert law, *Q* can be written as

(2) $$Q(x,y,z,t)=-\alpha I(x,y,t)(1-R)e^{-\alpha z},$$

where *α* is the absorption coefficient of the material, *R* is the reflectivity of the sample, and *I*(*x*, *y*, *t*) is the intensity distribution of the laser. The spatial distribution of *I*(*x*, *y*, *t*) was assumed to have a Gaussian shape for GLA and a rectangular shape for BLA in the *xy* plane according to the actual spatial distribution of each laser. The temporal distribution of *I*(*x*, *y*, *t*) for GLA was also assumed to have a Gaussian shape with the full width half maximum (FWHM) of 30 ns based on the measured pulse shape of the laser. In addition, the following equations were used for the convective cooling and radiation conditions:

(3) $$n\cdot (\kappa \nabla T)=h(T_{\mathrm{amb}}-T)\;\mathrm{and}\;n\cdot (\kappa \nabla T)=\varepsilon k(T_{\mathrm{amb}}^{4}-T^{4}),$$

where *h*, *T*_amb_, *ε*, and *k* are the convection constant, ambient temperature of the processing atmosphere, thermal emission coefficient, and Boltzmann constant, respectively. **n** is the normal vector toward exterior of the boundary. *T*_amb_ was set at 25 °C in simulations, and the substrate temperature of samples was also set to be the same as *T*_amb_. For the value of *h*, we chose 10 W/m^2^K, which roughly corresponds to *h* of air. However, the relative contribution of the convection and radiation to the temperature distribution inside the sample was found to be <0.5%. The material parameters used for the simulation are listed in Table 1, which were adopted from Refs. [31,32,33,34].

In Reference [30], thermal simulation for laser annealing was also performed using COMSOL Multiphysics and the temperature distribution for one specific power condition was presented. Compared to the results in Reference [30], in this work, the thermal simulation was performed more systematically for BLA and GLA, including temporal and spatial distributions, and the temperature distributions for different power levels were investigated to find the optimum power condition.

### 2.2. Annealing Experiment

The laser annealing equipment was constructed to perform GLA and BLA. The *a*-Si sample for annealing was placed on a computer-controlled *x*-*y* stage. The sample dimension for the annealing experiment was about 1 × 1 cm^2^. The temperature of the sample stage was the same as the ambient temperature of 25 °C. In the laser annealing experiment, a laser beam was focused on the sample surface using an objective lens and it scanned the sample surface in the *xy* plane by moving the sample stage, as shown in Figure 1. The scanning speed was chosen to be 4 cm/s, which was expected to generate a reasonably uniform temperature distribution in the scanning direction. For the 532-nm laser in GLA, the second harmonic of a *Q*-switched DPSS Nd:YAG laser was employed. The pulse repetition rate and pulse width were 30 kHz and 30 ns, respectively. When the laser was illuminated on the sample surface, the beam profile was approximately measured to be a Gaussian function with the beam waist of ~6 μm. The CW laser for BLA consisted of two GaN-based blue laser diodes emitted at 450 nm. The laser beam of BLA has a rectangular shape with a spot size of 6 μm × 2 μm. The laser power was monitored using a laser power meter during the annealing process. The laser annealing experiments were performed under nitrogen environment. The sample for laser annealing was placed inside a small plastic container, where the nitrogen gas was filled. The pressure of nitrogen was maintained at 1.5 times the atmospheric pressure.

For GLA, the average power of lasers was measured from the power meter, and the peak power was determined using the pulse width and pulse repetition rate. The laser annealing experiments were mainly performed at the peak power of 2.7 W for GLA and a CW power of 530 mW for BLA. At these laser powers, the temperature at the sample surface reached ~2900 K based on the thermal simulation result, which will be shown later. Assuming the Gaussian shape in the spatial and temporal distribution of pulsed lasers, the peak power of 2.7 W for GLA corresponds to the pulse energy of 86 nJ and energy density of 153 mJ/cm^2^. In addition to GLA and BLA, thermal furnace annealing was performed to compare the crystallinities of the laser-annealed poly Si samples. In the furnace annealing experiment, the *a*-Si sample was annealed for 30 min at 800 °C.

**Figure 2:**
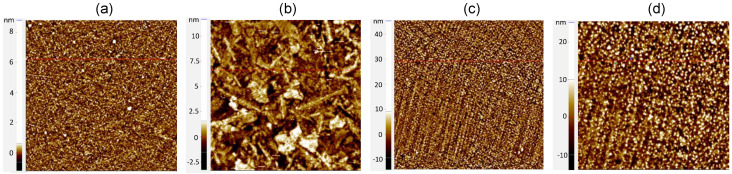
AFM images of a sample area of 5 × 5 μm^2^ for (**a**) an *a*-Si sample before annealing, (**b**) a furnace-annealed poly Si sample, (**c**) a poly Si sample annealed via BLA, and (**d**) a poly Si sample annealed via GLA.

Figure 2 shows atomic force microscopy (AFM) images of an *a*-Si sample before annealing, poly Si samples annealed via furnace annealing, BLA, and GLA. The AFM images were measured on a sample area of 5 × 5 μm^2^. The root-mean-square (RMS) roughness of the samples was determined from the AFM images in Figure 2. The RMS roughness was 0.3, 1.2, 4.5, and 5.7 nm for *a*-Si before annealing, furnace annealing, BLA, and GLA, respectively.

## 3. Results and Discussion

### 3.1. Thermal Simulation Results

**Figure 3:**
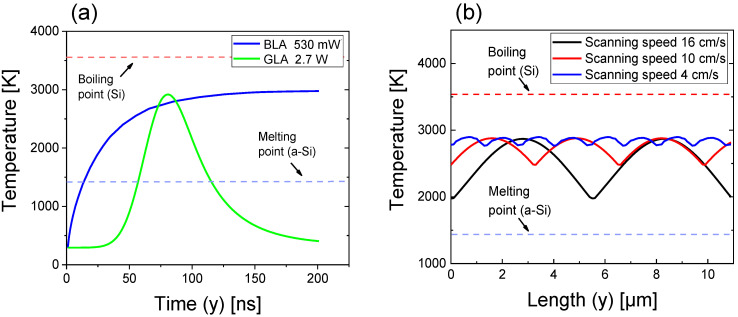
(**a**) Temperature on the sample surface as a function of time for BLA with the CW power of 530 mW and GLA with the peak power of 2.7 W. (**b**) Temperature profiles on the sample surface in the laser scanning direction (*y* direction in Figure 1) for GLA with the peak power of 2.7 W when the scanning speed is 4, 10, and 16 cm/s.

Using Equations (1)–(3) and the material parameters in Table 1, 3-D thermal simulations were performed to obtain temperature distributions in the *xy* plane and in the *z* direction as the laser power varied. The simulation area was 20 × 20 μm^2^, which is sufficiently larger than actual laser beam spot area. Figure 3a shows the temporal variation of the temperature at a certain point on the sample surface as the laser beam scans the sample. The temperature is plotted as a function of time for GLA with the peak power of 2.7 W and BLA with the CW power of 530 mW. These laser powers correspond to the maximum surface temperature of ~2900 K. For GLA, the temperature increased rapidly and reached a peak value, and then it decreased to the ambient temperature. This temporal response of the temperature resulted from the pulsed operation of the 532-nm laser. For BLA, on the contrary, the temperature increased slowly with time and reached a constant value because of the CW operation of the 450-nm laser.

Figure 3b shows the surface temperature of the *a*-Si layer as a function of the sample length in the scanning direction (the *y* direction in Figure 1) for three different scanning speeds of GLA. The temperature in Figure 3b corresponds to the maximum temperature in the temporal variation of temperature in Figure 3a. Here, the peak power of the 532-nm green laser was set to 2.7 W. When a pulsed laser scans the sample surface, the temperature in the scanning direction becomes inhomogeneous, depending on the pulse duration and repetition time. The temperature difference increased with the increase in the scanning speed, as shown in Figure 3b. When the scanning speed was 16 cm/s, the highest and lowest temperatures on the sample surface were 2870 and 1980 K, respectively. The difference in the surface temperatures was ~900 K, which could result in a significant non-uniformity in the crystallinity. At a scanning speed of 10 cm/s, the temperature difference was ~400 K, which was less than half of that at a scanning speed of 16 cm/s. When the scanning speed was further decreased to 4 cm/s, the surface temperature varied from 2780 to 2900 K. That is, the temperature difference was reduced to 120 K, which is expected to yield a considerably improved uniformity in the crystallinity. From the result of Figure 3b, it is expected that the crystallinity of the laser-annealed poly Si film is improved as the scanning speed decreases, at the expense of a long laser processing time. In this study, we selected 4 cm/s as the scanning speed of GLA to obtain uniform crystallinity. For BLA, the temperature in the scanning direction was found to be almost uniform because of the CW operation of blue lasers.

**Figure 4:**
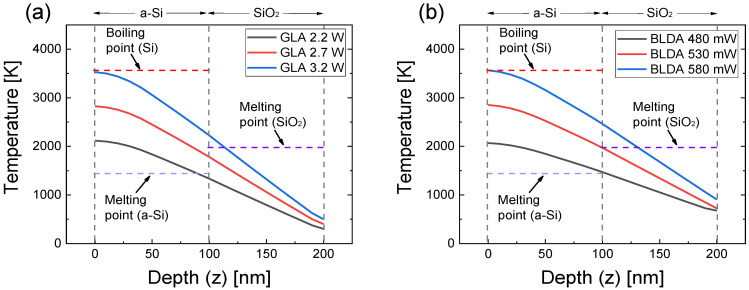
Simulated temperature profile in the direction of the sample depth (the *z* direction in Figure 1) for (**a**) GLA with the laser peak powers of 2.2, 2.7, and 3.2 W and (**b**) BLDA with the CW laser powers of 480, 530, and 580 mW. Here, the maximum temperature in the temporal profile in Figure 3a is plotted at each *z* point.

Figure 4 shows the simulated temperature distributions as a function of the sample depth (the *z* direction in Figure 1) for three different power levels of GLA and BLA. The temperature decreased steadily with increasing depth. The temperature at the interface between *a*-Si and SiO_2_ should be higher than the melting point of *a*-Si (1420 K) for crystallizing the *a*-Si layer. However, the temperature on the surface of the *a*-Si layer should be lower than the boiling point of silicon (3538 K) to avoid the vaporization of the annealed poly Si. In addition, the temperature of the underlying SiO_2_ layer should be lower than the melting point of SiO_2_ (1986 K) to protect it from thermal damage. Therefore, the optimum power condition should be chosen such that the temperature on the sample surface is lower than the boiling point of Si, and the temperature at the *a*-Si/SiO_2_ interface is higher than the melting point of *a*-Si and lower than the melting point of SiO_2_.

In Figure 4, the surface temperature corresponded to the boiling point of Si for the laser power of 580 mW for BLA and 3.2 W for GLA. For the laser powers of 530 mW for BLA and 2.7 W for GLA, the surface temperature was approximately 2900 K, which was significantly higher than the melting point of *a*-Si and sufficiently lower than the boiling point of Si. For these laser powers, the temperature at the *a*-Si/SiO_2_ interface was well above the melting point of *a*-Si and below the melting point of SiO_2_ for both BLA and GLA. Therefore, we believe these laser powers could be close to the optimum condition for achieving good crystallinity. When the laser power decreased to 480 mW for BLA and 2.2 W for GLA, the surface temperature was ~2000 K, which was still higher than the melting point of *a*-Si. However, for these laser powers, the temperature at the *a*-Si/SiO_2_ interface was just similar to the melting point of *a*-Si, which could result in an incomplete crystallization of *a*-Si.

### 3.2. Raman Spectoscopy

**Figure 5:**
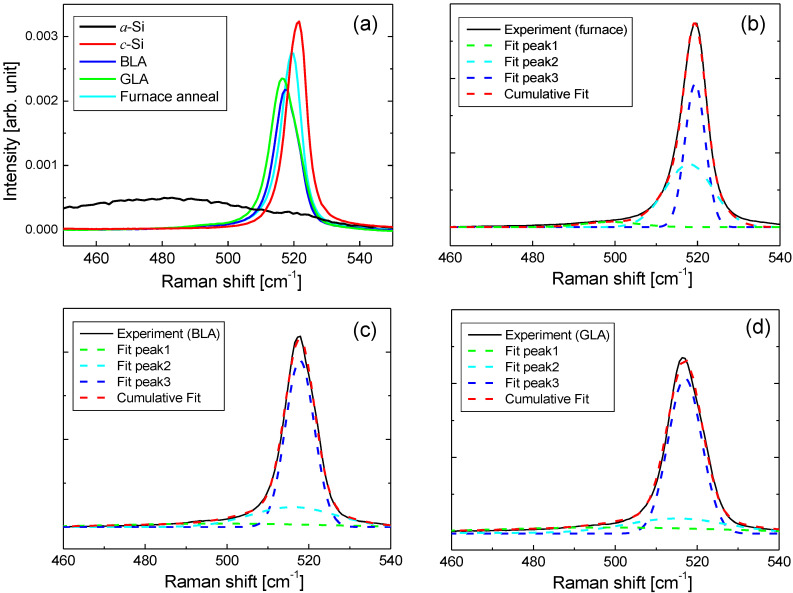
(**a**) Raman spectra of *a*-Si, *c*-Si, and poly silicon samples with BLA, GLA, and thermal furnace annealing. (**b**–**d**) Fit of Raman spectra of poly Si films formed via furnace annealing, GLA, and BLA, respectively. The experimental spectrum is represented by the black solid lines. The green, cyan, and blue dotted lines represent the fitted three Gaussian curves. The red lines represent the cumulative fit curves.

After the annealing processes, the crystallinity of the annealed poly Si was investigated using Raman spectroscopy and spectroscopic ellipsometry. Raman spectroscopy has been widely employed for investigating the crystal quality of poly Si [28,29,30]. Raman spectrum for each sample was measured using a micro Raman system (UniDRON-3200, UniNano Tech Co., Yongin-si, Korea). In the Raman spectroscopy system, a CW 532-nm laser with a power of 25 mW and a spot size of ~3 μm was adopted. The resolution of Raman shift was 0.5 cm^−1^. Figure 5a shows the Raman spectra of annealed poly Si samples along with those of *a*-Si and crystalline silicon (*c*-Si) samples. The Raman spectrum of *a*-Si shows a broad spectral distribution centered at approximately 480 cm^−1^ associated with transverse optic (TO) phonons. In contrast, *c*-Si shows a sharp peak in the TO phonon mode at approximately 520 cm^−1^. The crystallinity of the annealed poly Si film can be analyzed using the peak shift and FWHM of *a*-Si and *c*-Si. As the crystallinity improves, the peak in the TO phonon mode of poly Si shifts towards that of *c*-Si. The peak Raman shift for the furnace annealing, BLA, and GLA was 519, 517.5, and 516.5 cm^−1^, respectively. The FWHM for the furnace annealing, BLA, and GLA was 7.5, 9.0, and 9.5 cm^−1^, respectively. Therefore, from the Raman spectra in Figure 5a, it is expected that the furnace-annealed poly Si sample will show better crystallinity than the laser-annealed poly Si samples, and BLA will show slightly better crystallinity than GLA.

Recently, the crystal volume fraction (*f_c_*) of poly Si was evaluated using the relative values of FWHM and intensity using TO phonon mode of the materials in the Raman spectrum [35]. *f_c_* of poly Si can be quantitatively evaluated using the following formula:

(4) $$f_{c}=\frac{I_{c}}{I_{c}+I_{a}},$$

where *f_c_* represents the crystalline volume fraction. *I_c_* and *I_a_* indicate the integrated Raman scattering intensity of crystalline and amorphous sections, respectively. *I_c_* and *I_a_* can be obtained by the deconvolution of each Raman spectrum into Gaussian components corresponding to the crystalline and amorphous phases, respectively.

Figure 5b–d shows the fit of Raman spectra of poly Si films formed via furnace annealing, GLA, and BLA, respectively. In this case, the peak power of GLA was 2.7 W and the CW power of BLA was 530 mW. Each Raman spectrum was decomposed into three Gaussian functions corresponding to one amorphous phase and two crystalline parts of a weak crystalline phase and a full crystalline phase [35]. According to Ref. [35], the two crystalline phases can be attributed to nanocrystalline silicon and crystalline silicon phases, respectively. The peak positions of three Gaussian functions centered around 498, 516, and 518 cm^−1^. In Figure 5, the Gaussian functions of the peaks 1 and 2 correspond to *I_c_* and that of the peak 3 corresponds to *I_a_* in Equation (4), respectively. The measured Raman spectrum (black solid line) and the cumulative fit curve (red dotted line) of the three Gaussian functions show good consistency for all cases. As *a*-Si becomes crystallized to poly Si, the TO phonon peak of the crystalline phase is shifted slowly toward the peak of *c*-Si and the FWHM is decreased, resulting in an increase in *f_c_*. From the fitting results, *f_c_* was calculated using Equation (4). The *f_c_* value of poly Si formed via furnace annealing was 96.2%, which was the highest value among all annealed samples. The *f_c_* values for BLA and GLA were 90.6% and 88.2%, respectively, implying that the poly Si formed via BLA showed slightly higher crystal quality than that formed via GLA. This is consistent with the observation that BLA showed the longer Raman peak shift and the narrower FWHM than GLA as shown in Figure 5a. The crystal volume fraction values relative to furnace annealing were 94.1% and 91.7% for BLA and GLA, respectively, indicating that the laser-annealed poly Si showed crystallinity comparable to thermal furnace-annealed poly Si.

**Figure 6:**
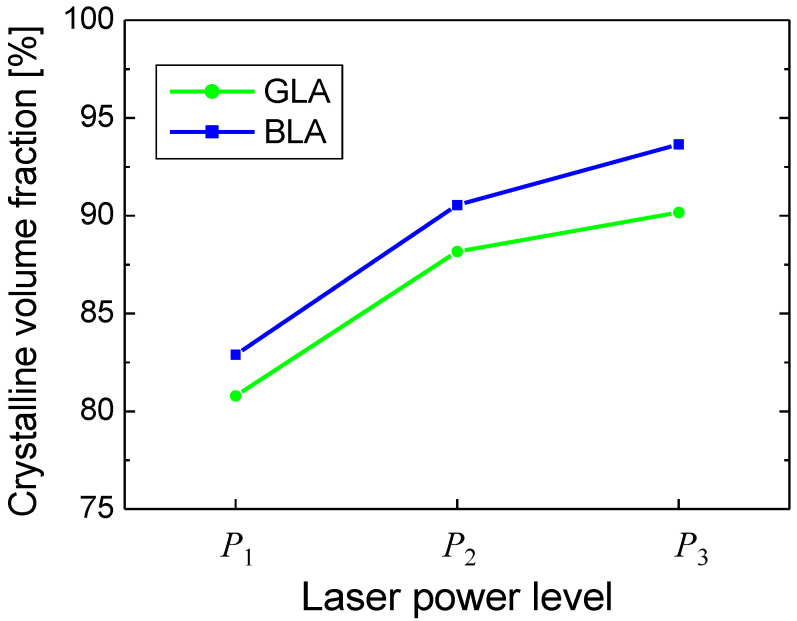
Crystal volume fraction (*f_c_*) for BLA and GLA at the three laser power levels shown in Figure 4. The laser power levels *P*_1_, *P*_2_, *P*_3_ respectively correspond to 480, 530, 580 mW for BLDA and 2.2, 2.7, 3.2 W for GLA.

To investigate the effect of laser power levels on crystallinity, laser annealing experiments were performed at other laser power levels that corresponded to the laser powers of thermal simulations in Figure 4. Then, the *f_c_* values were determined for each power level using the Raman spectrum analysis. In Figure 6, the *f_c_* values obtained by the Raman spectrum analysis are plotted. The three power levels *P*_1_, *P*_2_, *P*_3_ respectively correspond to 2.2, 2.7, 3.2 W for GLA and 480, 530, 580 mW for BLA. As the laser power increased from *P*_1_ to *P*_3_, *f_c_* increased from 80.8% to 90.2% for GLA and from 82.9% to 93.7% for BLA. For all laser power levels, BLA showed slightly higher *f_c_* values than GLA. The result in Figure 6 shows that the crystallinity of laser-annealed poly Si was improved as the laser power increased. At *P*_3_, BLA and GLA resulted in *f_c_* of >90%, which is close to *f_c_* of the thermal furnace annealing. However, it was found that surface roughness increased significantly at *P*_3_, implying partial boiling of Si might occur at a power level this high.

### 3.3. Spectroscopic Ellipsometry

The crystallinities of the annealed poly Si samples were also analyzed quantitatively using the optical constant spectra obtained by spectroscopic ellipsometry measurements. For the measurement, we employed a spectroscopic ellipsometer (V-VASE, JAWoollam Co., Lincoln, NE, USA) at a fixed angle of 70° in a wavelength range of 300 to 1600 nm. In ellipsometry, the amplitude and phase difference between two polarization states, which is often denoted as Ψ and Δ, are measured for the light reflecting from the surface of a thin film. The refractive index (*n*) and extinction coefficient (*k*) spectra can be obtained by the fit of experimental Ψ and Δ spectra. The effective medium approximation (EMA) method was employed for the fitting [36,37].

For the EMA process, a poly Si film was assumed to be composed of *a*-Si and *c*-Si. The crystalline volume fraction of the annealed poly Si can be obtained using the EMA method. Among the EMA methods, the Bruggeman-type method has been widely employed to estimate the volume fraction of composite materials for dielectric multilayers, and can be expressed as [36,37]

(5) $$f_{a}\frac{\varepsilon_{a}-\varepsilon_{poly}}{\varepsilon_{a}+2\varepsilon_{poly}}+f_{c}\frac{\varepsilon_{c}-\varepsilon_{poly}}{\varepsilon_{c}+2\varepsilon_{poly}}=0\;\mathrm{and}\;f_{a}+f_{c}=1,$$

where *ε_a_* and *ε_c_* are the dielectric functions of *a*-Si and *c*-Si, respectively. *ε_poly_* is an effective dielectric function of the composite material (poly Si). The dielectric function can be correlated with the complex refractive index, which is composed of the refractive index and extinction coefficient. *f_a_* and *f_c_* are the volume fractions of *a*-Si and *c*-Si, respectively. 

**Figure 7:**
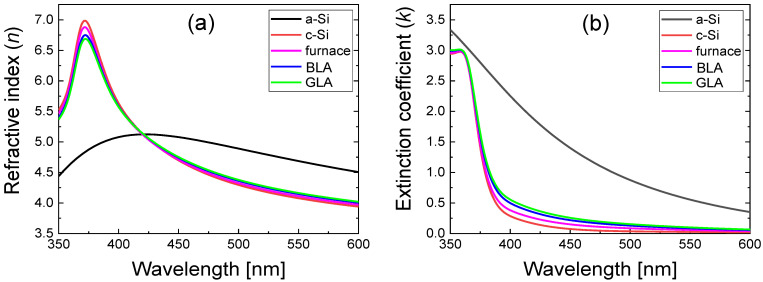
Optical constant spectra of poly Si samples annealed via furnace annealing, BLA, and GLA. The spectra of *a*-Si and *c*-Si are also shown for reference. (**a**) Refractive index (*n*) spectra and (**b**) extinction coefficient (*k*) spectra.

For fitting the experimental Ψ and Δ data, *f_c_* and the film thickness (*d*) were used as fitting parameters. The fitting results showed that the mean squared error (MSE) was 1~2. This MSE value is much smaller than previous results for similar thin film structures [35,38], indicating excellent data fitting with reliable results on *f_c_* and *d*. From the results of the fitting, the *f_c_* values of the annealed poly Si were determined to be 88.6%, and 85.6% for BLA and GLA, respectively. Using the obtained *f_c_* value, the *n*, *k* spectra of annealed poly Si can be obtained from Equation (5). Figure 7 shows *n* and *k* spectra of the laser-annealed poly Si samples, *c*-Si, and *a*-Si when the peak power of GLA was 2.7 W and the CW power of BLA was 530 mW. As the crystallinity improves, the spectral shape of poly Si approaches that of *c*-Si. It can be observed that the *n* and *k* spectra of the furnace-annealed poly Si are very close to those of *c*-Si, indicating the good crystal quality of the furnace-annealed sample. The laser-annealed poly Si samples also exhibit spectral shapes similar to those in the case of *c*-Si.

**Figure 8:**
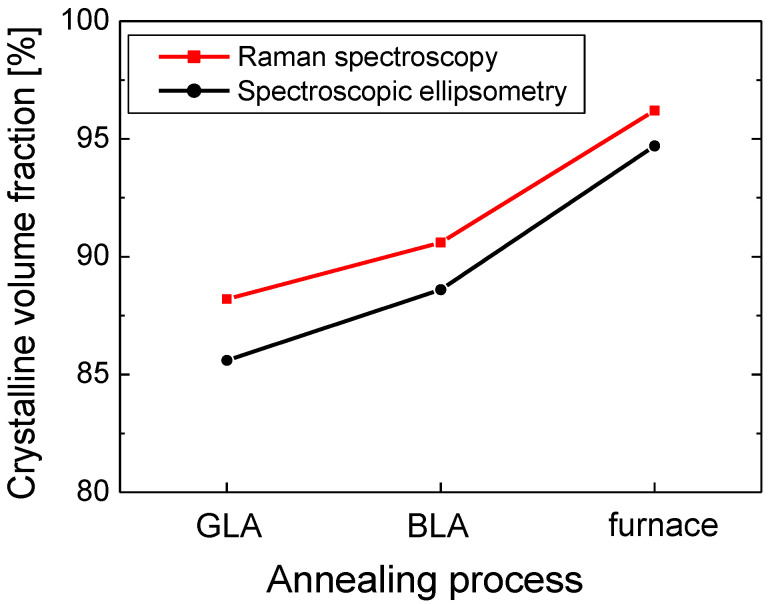
Crystalline volume fraction (*f_c_*) for GLA, BLA, and furnace annealing analyzed using Raman spectroscopy and spectroscopic ellipsometry.

In Figure 8, the *f_c_* values obtained by Raman spectroscopy and spectroscopic ellipsometry are compared for poly Si samples annealed via furnace annealing, GLA, and BLA when the laser power was 2.7 W for GLA and 530 mW for BLA. The results of the two methods show good correlation with a difference of only 2–3% in the *f_c_* value for all poly Si samples. The *f_c_* values obtained by spectroscopic ellipsometry were slightly lower than those obtained by Raman spectroscopy. The ellipsometry measurement also showed that the poly Si formed via BLA had slightly better crystal quality than that formed via GLA similarly to the case of Raman spectroscopy. The results of Figure 8 indicate that the Raman spectra as well as the *n* and *k* spectra obtained using ellipsometry can be successfully applied for the quantitative evaluation of the crystallinity of poly Si. The average *f_c_* values were 95.4%, 89.6%, and 86.9% for furnace annealing, BLA, and GLA, respectively.

The analysis results of this study showed that BLA could be better laser annealing process than GLA for the annealing of a 100-nm-thick *a*-Si layer, which is attributed to the CW laser operation in BLA. Owing to the CW operation, good spatial uniformity is expected. However, thermal damage in the underlayers, which might occur during the CW laser annealing, should be considered. As shown in the simulation result in Figure 4b, high temperatures of ~730 K could be maintained even below the SiO_2_ underlayer. Although GLA showed slightly lower *f_c_* than BLA, it also resulted in a good crystallinity with *f_c_* > 85%. At the interface between the SiO_2_ underlayer and the Si substrate, the temperature of GLA was 400 K, indicating thermal damage to the substrate material could be avoided. In addition, despite the pulsed operation, a reasonably good spatial uniformity is also expected from the simulation results in Figure 3 if the scanning speed is sufficiently slow. The quantitative analysis results presented in this paper imply that the optimum laser-annealing process is expected to realize poly Si with high crystallinity suitable for LTPS process.

## 4. Conclusions

In summary, we quantitatively evaluated the degree of crystallization of poly Si thin films annealed via GLA with a 532-nm nanosecond laser and BLA with 450-nm CW laser by determining the crystal volume fraction of annealed poly Si using Raman spectroscopy and spectroscopic ellipsometry. 3-D heat transfer simulations were performed to obtain the temperature distributions during laser annealing processes, and GLA and BLA experiments were conducted based on the thermal simulation results. The *f_c_* values of annealed poly Si were determined by fitting of the Raman spectrum and the optical constant spectra obtained by ellipsometry. Both the Raman spectroscopy and ellipsometry showed consistent result on the *f_c_* values. It was found that the evaluated *f_c_* values of laser-annealed poly Si could be as high as 90%, which was comparable to the *f_c_* of thermal furnace-annealed poly Si, implying good crystallinity of the laser-annealed poly Si thin films. Because of its simplicity, Raman spectroscopy is expected to be advantageously employed for evaluating the crystal quality of laser-annealed poly Si.

## Figures and Tables

**Table 1:** Material parameters used for thermal simulation.

Parameter	Type	Value
Density (*ρ*)	*c*-Si	2329 kg/m^3^
	*a*-Si	2260 kg/m^3^
	SiO_2_	2203 kg/m^3^
Melting temperature	*a*-Si	1420 K
	SiO_2_	1986 K
Heat capacity (*C_p_*)	*c*-Si	700 J/(kg·K)
	*a*-Si	992 J/(kg·K)
	SiO_2_	703 J/(kg·K)
	Melted Si	965 J/(kg·K)
Thermal conductivity (*κ*)	*c*-Si	130 W/m·K
	*a*-Si	1.5 W/m·K
	SiO_2_	1.38 W/m·K
	Melted Si	2 W/m·K
Reflectivity (*R*)	450-nm laser	0.483 (a-Si), 0.7(Melted Si)
	532-nm laser	0.403 (a-Si), 0.71 (Melted Si)
Surface emissivity coefficient (*ε*)	*a*-Si	0.5
	Melted Si	0.7
Latent heat	*a*-Si	1789.42 J/g
Convection constant (*h*)	air	10 W/m^2^K
Absorption coefficient (*α*)	450-nm laser	0.0436 nm^-1^
	532-nm laser	0.0207 nm^-1^

## Data Availability

The data supporting the findings of this paper is available from the corresponding authors upon reasonable request.
